# Genome-wide analysis of annexin gene family in *Schrenkiella parvula* and *Eutrema salsugineum* suggests their roles in salt stress response

**DOI:** 10.1371/journal.pone.0280246

**Published:** 2023-01-18

**Authors:** Fatemeh Moinoddini, Amin Mirshamsi Kakhki, Abdolreza Bagheri, Ahmad Jalilian

**Affiliations:** Department of Biotechnology and Plant Breeding, Faculty of Agriculture, Ferdowsi University of Mashhad, Mashhad, Iran; Bhabha Atomic Research Centre, INDIA

## Abstract

Annexins (Anns) play an important role in plant development, growth and responses to various stresses. Although *Ann* genes have been characterized in some plants, their role in adaptation mechanisms and tolerance to environmental stresses have not been studied in extremophile plants. In this study, *Ann* genes in *Schrenkiella parvula* and *Eutrema salsugineum* were identified using a genome-wide method and phylogenetic relationships, subcellular distribution, gene structures, conserved residues and motifs and also promoter prediction have been studied through bioinformatics analysis. We identified ten and eight encoding putative *Ann* genes in *S*. *parvula* and *E*. *salsugineum* genome respectively, which were divided into six subfamilies according to phylogenetic relationships. By observing conservation in gene structures and protein motifs we found that the majority of *Ann* members in two extremophile plants are similar. Furthermore, promoter analysis revealed a greater number of GATA, Dof, bHLH and NAC transcription factor binding sites, as well as ABRE, ABRE3a, ABRE4, MYB and Myc cis-acting elements in compare to *Arabidopsis thaliana*. To gain additional insight into the putative roles of candidate *Ann* genes, the expression of *SpAnn*1, *SpAnn*2 and *SpAnn*6 in *S*. *parvula* was studied in response to salt stress, which indicated that their expression level in shoot increased. Similarly, salt stress induced expression of *EsAnn*1, 5 and 7, in roots and *EsAnn*1, 2 and 5 in leaves of *E*. *salsugineum*. Our comparative analysis implies that both halophytes have different regulatory mechanisms compared to *A*. *thaliana* and suggest *SpAnn2* gene play important roles in mediating salt stress.

## Introduction

Salinity is one of the most important abiotic stresses that affects the growth and crop yields, significantly. Soil salinity affects around 50% of arable land worldwide [1], while most food crops are salt sensitive (glycophyte), which poses a threat to global food security. Halophyte-based researches can provide valuable information to improve abiotic stress tolerance in crops. Halophytes are salt-tolerant plants that can complete their life cycle in salt concentration of 200 mM NaCl or more [2]. There are 18 halophytes in the Brassicaceae family, including *Schrenkiella parvula* (earlier called *Thellungiella parvula*) and *Eutrema salsugineum* (formerly known as *Thellungiella salsuginea*), which deploy multiple adjustments under salt stress [3]. To cope with high salt concentration, Halophytes have evolved mechanisms such as dormancy-like states to salt inhibit germination, improved ion homeostasis, morphological features (e.g., higher stomatal density and a second layer of endodermis), an up-regulated antioxidant system and also "stress ready" transcriptome and metabolome [4].

Annexin (Ann) is a Ca^2+^ and membrane-binding protein which is reported to be involved in plant growth, development and various stress responses such as salt stress [5]. The *Ann* genes have already been discovered in Brassica species such as *A*. *thaliana* [6], *B*. *juncea* [7], *B*. *rape* [8], *B*. *oleracea* [9], *B*. *napus* [9] and other plants like *Oryza sativa* [10], *Triticum aestivum* [11], *Zea mays* [12], *Solanum lycopersicum* [13]. A multi-gene and multi-functional family of Ann proteins are involved in important biological activities, including ion transport, cellular homeostasis, membrane trafficking and cytoskeletal organization [14]. Anns as Ca^2+^ binding proteins, act as Ca^2+^ signal transducers. Laohavisit et al. [15], demonstrated that *A*. *thaliana* Annexin1 (AtAnn1) as a ROS-activated plasma membrane Ca^2+^ channel, is one of the candidate channel that involves in mediating [Ca^2+^]cyt accumulation under salinity stress. Apart from AtAnn1, Huh et al. [16] showed that AtAnn4 also interact with AtAnn1 and regulate growth and viability under saline and drought conditions. In rice the expression levels of *OsAnn6* and *OsAnn7* increased in response to salt stress, while the expression levels of *OsAnn1* and *OsAnn10* decreased significantly [10]. Yadav et al. [8], discovered that the transcript levels of *Anns* (*BraAnn*1,2,4 and 8) in *Brassica rapa* increased under salt stress.

Several functional analyses have revealed that *Ann* genes have a positive effect on plant stress tolerance. *AtAnn1* gain-of-function mutants in *A*. *thaliana* were more drought tolerant than *AtAnn1* loss-of-function mutants [17]. In rice, overexpressing of *OsAnn3* reduces water loss by enhancing root length and stomata closure in an ABA-dependent manner and accordingly confers stress tolerance [18]. Also, overexpression of *StAnn1* in potato increased drought tolerance in addition to modifying redox state and phytohormone mediated pathways [19]. In addition, overexpression of *Ann2* from *Solanum pennellii* improved salt and drought tolerance in *Solanum lycopersicum* [20].

The regulatory mechanisms of plant *Anns* in abiotic stress have been investigated in several studies. In Arabidopsis, under salt stress the SOS2-SCaBP8 complex generates and fine-tunes an *AtAnn4*-dependent calcium signature [21]. Also, cold-activated OST1/SnRK2.6 phosphorylates *AtAnn1* and enhances its Ca^2+^ transport activity which generates a Ca^2+^ signal that mediates freezing tolerance [22]. In *A*. *thaliana*, cryptochrome 2 represses the functions of *AtAnn2* and *AtAnn3* by affecting their subcellular localization and transmembrane Ca^2+^ flow in drought stress [23].

However, there has not been any report on *Anns* in extremophiles species. A comparative study of the *Ann* gene family in halophyte models versus *A*. *thaliana* as a glycophyte would provide a starting point for understanding how the *Ann* gene family contributes to the halophyte’s salt tolerance. In this study, *Ann* genes were identified in two halophytes of *S*. *parvula* and *E*. *salsugineum* through genome-wide analysis. We compared *SpAnn* and *EsAnn* gene’s structures, conserved motifs and promoter region with *A*. *thaliana*’s *Ann* genes to investigate their roles in the salt tolerance of the halophytes. Furthermore, the expression of chosen *Ann* genes in *S*. *parvula* and also *E*. *salsugineum* in response to salt stress were investigated. Our findings suggest the roles of *SpAnn2* gene in mediating salt stress and provide valuable information for further study on the function of *Ann* genes in halophyte growth, development and stress responses.

## Materials and methods

### Plant materials and NaCl treatment

Seeds of *S*. *parvula* (Lake Tuz ecotype) were sown on a 7:2:1 soil mixture (peat moss/vermiculite/perlite) and stratified for 7 days at 4°C in dark condition. Then, the seeds were grown at 22/20°C (day/night) with a relative humidity of 60% and a photoperiod of 16 /8 h (light/dark) at light intensity 130 μmol m^-2^ s^-1^. Three weeks after germination (vegetative development), plants were treated with a 200 mM NaCl solution and water as a control. The shoots of treated and non-treated plants were collected at 3 h, 6 h and 12 h after NaCl treatment. Liquid nitrogen was used to freeze all samples before storing at -80°C for future use.

### Identification and phylogenetic analysis of Ann family members

The TAIR database (http://www.arabidopsis.org/) was utilized to get the Ann protein sequences of *A*. *thaliana*. The sequence protein of *S*. *parvula* and *E*. *salsugineum* were obtained from phytozome v13 (https://phytozome-next.jgi.doe.gov/), EnsemblPlants (https://plants.ensembl.org/index.html/) and Thellungiella (http://thellungiella.org/) by the BLASTP program in which E-value cut-off of 1E-5 were applied [24]. Furthermore, sequence identity >75% and similarity >80% were considered as threshold to determine homology. Finally, sequences with low degrees of similarity, poor domain coverage and proteins with different functional annotations were filtered. The ProtParam tool from Expasy (http://us.expasy.org/tools/protparam) was applied to physiochemically describe the investigated proteins with default settings. Also, to predict protein subcellular localization, the CELLO online program v2.5 was used (http://cello.life.nctu.edu.tw/) [25].

The protein sequences of the *Ann* gene family in *A*. *thaliana*, *S*. *parvula*, *E*. *salsugineum* and also *B*. *napus* and *B*. *rapa* as Brassicaceae species, were aligned by the ClustalW program of MEGA-X [26] by default settings. The bootstrap value for the neighbor-joining method of MEGAX, which was used to construct the phylogenetic trees, was set to 1000 [10].

### Gene structure and conserved motif analysis

In order to prediction of the exon and intron structures of *Ann* genes, the Gene Structure Display Server 2.0 (GSDS v2.0) was used (http://gsds.gao-lab.org/) [27]. Conserved motifs/residues in *A*. *thaliana*, *S*. *parvula* and *E*. *salsugineum* have been identified using multiple sequence alignment (MSA) generated by the ClustalW program of MEGA-X [26] and illustrated by of ESPript v3.0 (https://espript.ibcp.fr) [28].

Conserved protein motifs were analyzed using the MEME (https://meme-suite.org/meme/tools/meme) with the number of different motifs set to 5. For the distribution of motifs among the sequences, any number of repetitions (anr) was selected [29]. InterProScan (https://www.ebi.ac.uk/interpro/) was used to annotate motifs [30]. To verify the domains in Ann protein sequences of *A*. *thaliana*, *S*. *parvula* and *E*. *salsugineum*, the SMART online program (http://smart.embl-heidelberg.de/) was used [31].

### Cis-acting elements and transcription factor binding sites analysis

The PlantCARE (http://bioinformatics.psb.ugent.be/webtools/plantcare/html/) was applied to look for cis-elements in the 2 Kb upstream of the 5′-UTR of *A*. *thaliana*, *S*. *parvula* and *E*. *salsugineum Ann* genes [32]. The promoter regions were divided into three groups: proximal, median and distal, which were 500, 501–1000 and 1001–1500 bp upstream, respectively. To identify the putative transcription factor binding sites (TFBSs) on promoters, the TF Binding Site Prediction program in the PlantRegMap (http://plantregmap.gao-lab.org/binding_site_prediction.php) was used with a threshold p-value ≤ 1e-6 (apart from *SpAnn7* with 381 bp upstream) [33]. PANTHER (http://pantherdb.org/) was used to find the Gene Ontology biological process analysis of the three plants [34].

### Nonsynonymous/ Synonymous substitution ratio (Ka/Ks)

The nonsynonymous (Ka) and synonymous (Ks) substitution rates and the Ka/Ks value between homologous gene pairs were calculated using DnaSP v6 [35].

### Gene expression analysis of the *Ann* genes under salt stress

A total RNA extraction Mini Kit (Favorgen®) was used to extract total RNA from the shoots of three-week-old plants (4 to 6 true leaves). RNase-Free DNase I (Yekta Tajhiz Azma®) was used to remove DNA contamination from RNA samples according to the manufacturer’s instructions. The Easy cDNA Synthesis Kit (Parstous®) was used to synthesize first-strand cDNA from two μg of total RNA. The reactions were performed using the RealQ Plus 2x Master Mix Green (Ampliqon®) in a qRT-PCR system (CFX96 Dx Real-Time PCR Detection Systems (Bio-Rad)) according to the instructions given by the manufacturer. The primers are listed in S5 Table. Actin 7 was selected as an internal control gene, for all analyses [36]. Three biological replicates with six plant samples each were tested, along with two technical replicates. The 2^−ΔΔCt^ method was used to compute the relative quantification of various mRNA levels based on the cycle threshold (Ct) [37]. The expression data for *Ann* genes in *E*. *salsugineum* was retrieved from the public Short Read Archive (SRA) (SRP323931 and SRP135727) of NCBI to explore their levels of expression [38]. The expression data for candidate *Ann* genes in *S*. *parvula*’s root were acquired from Li et al. study [39]. The heatmap was performed using the GraphPad Prism v9.

### Statistical analysis

Three biological replicates were used in all experiments. Data were analyzed via GLM procedure of SAS software version 9.1 (SAS Institute 2003). In order to determine significant differences between mean of the treatments, Tukey’s multiple comparisons test (Tukey _0.05_) was applied.

## Results

### Identification and phylogenetic analysis of *Ann* gene family in *S*. *parvula* and *E*. *salsugineum*

Arabidopsis Ann (AtAnn) proteins were retrieved from the TAIR database and the BLASTP program was used against two databases (Phytozome and Thellungiella for *S*. *parvula*, Phytozome and Ensembl Plants for *E*. *salsugineum*) to identify putative homologous proteins. Ten and eight *Ann* genes were identified in *S*. *parvula* and *E*. *salsugineum*, respectively. Some basic properties of *Ann* genes are shown in Table 1. Protein Sequence comparison with *A*. *thaliana* Anns show high sequence identity (79–93% for SpAnns and 83–94% for EsAnns) and similarity (88–98% for SpAnns and 86–97% for EsAnns). *Ann3* in both halophytes have the longest CDS length (960 bp), while *SpAnn4-2* with 945 bp, and *EsAnn1* with 837 bp, have the shortest length of CDS. SpAnns protein have 314 to 319 amino acids (aa) with a molecular weight extended in the range of 35.74 to 36.64 kDa, and EsAnn proteins have 278 to 319 aa with a molecular weight extended in the range of 31.70 to 36.47 kDa. SpAnn and EsAnn proteins all have four annexin repeats except SpAnn4-1, SpAnn4-2 and also EsAnn4 which have two annexin repeats. Isoelectric points (pI) extended in the range of 4.91 to 9.56 and from 5.11 to 9.61 for SpAnns and EsAnns, respectively. SpAnns and EsAnns are present in different subcellular locations, including cytoplasm, nuclear and mitochondria. SpAnn2 and EsAnn7 are located in the cytoplasm while SpAnn4-1, SpAnn5 and EsAnn4 are located in the nuclear. Most of the SpAnns and EsAnns were localized in two subcellular organelles. SpAnn and EsAnn 1, 3, 8 as well as EsAnn2, SpAnn2-4 and SpAnn6 are found both in the cytoplasm and nuclear compartments. EsAnn5 is located in nuclear and mitochondria while EsAnn6 is located in the cytoplasm as well as mitochondria. *Ann* genes are unevenly located on five chromosomes (ch) and five scaffolds in *S*. *parvula* and *E*. *salsugineum*, respectively. *S*. *parvula* has four *Ann* genes on ch4 (SpAnn3-1, 3–2, 4–1 and 4–2) and two on ch6 (SpAnn6 and SpAnn8) while ch1, 2 and 5 each have only one *SpAnn* gene. Scaffold 2 and scaffold 10 with three (*EsAnn6*, *7* and *8*) and two (*EsAnn3* and *4*) respectively, contained the highest number of *Ann* genes in *E*. *salsugineum*. In contrast, scaffold 23, 6 and 9 each have only one *EsAnn* gene. As the *SpAnn7* gene is unlocated, the above-mentioned analyses were not considered for it (Table 1).

**Table 1:** List of *Ann* genes identified in *A*. *thaliana*, *S*. *parvula* and *E*. *salsugineum*.

Gene ID *A*. *thaliana*	Halophytes Models	Homologous in *S*. *parvula* / *E*. *salsugineum*	Gene name	ID*	SI*	CDS (bp) *	Size (AA) *	pI*	Mw (KD) *	Annexin repeats	Subcellular localization *	Chromosome location
AT1G35720 (Ann1)	*S*. *parvula*	Sp1g30430	*SpAnn1*	93%	98%	954	317	5.01	36.14	4	CYP, NUC	Ch1-1: 12102701..12104544
	*E*.*salsugineum*	Thhalv10001831m.g	*EsAnn1*	83%	86%	837	278	5.11	31.70	4	CYP, NUC	Scaffold_23: 1193422..1195415
AT5G65020 (ANN2)	*S*. *parvula*	Sp2g28100	*SpAnn2*	91%	96%	951	316	5.62	36.01	4	CYP	Ch2-4: 8656245..8657989
	*E*.*salsugineum*	Thhalv10004625m.g	*EsAnn2*	93%	97%	951	316	5.9	36.08	4	CYP, NUC	Scaffold_6: 1049857..1052136
AT2G38760 (ANN3)	*S*. *parvula*	Sp4g21120	*SpAnn3-1*	84%	91%	960	319	5.79	35.81	4	CYP, NUC	Ch4-6: 4968516..4970183
	*S*. *parvula*	Sp4g21140	*SpAnn3-2*	81%	89%	960	319	4.91	35.85	4	CYP, NUC	Ch4-6: 4975459..4976906
	*E*.*salsugineum*	Thhalv10016949m.g	*EsAnn3*	83%	92%	960	319	5.76	35.93	4	CYP, NUC	Scaffold_10: 9888744..9890377
AT2G38750 (ANN4)	*S*. *parvula*	Sp4g21110	*SpAnn4-1*	83%	92%	954	317	8.24	36.18	2	NUC	Ch4-6: 4964220..4966590
	*S*. *parvula*	Sp4g21130	*SpAnn4-2*	79%	88%	945	314	5.70	36.15	2	NUC, CYP	Ch4-6: 4970640..4972561
	*E*.*salsugineum*	Thhalv10016965m.g	*EsAnn4*	85%	93%	948	315	7.25	35.71	2	NUC	Scaffold_10: 9884450..9886721
AT1G68090 (ANN5)	*S*. *parvula*	Sp5g23090	*SpAnn5*	93%	97%	951	316	9.56	36.08	4	NUC	Ch5-6: 576885..578244
	*E*.*salsugineum*	Thhalv10019463m.g	*EsAnn5*	94%	97%	951	316	9.61	36.03	4	NUC, MIT	Scaffold_9: 6077152..6078532
AT5G10220 (ANN6)	*S*. *parvula*	Sp6g33180	*SpAnn6*	90%	94%	957	318	8.55	36.64	4	NUC, CYP	Ch6-6: 8411572..8414245
	*E*.*salsugineum*	Thhalv10014136m.g	*EsAnn6*	93%	96%	957	318	6.69	36.47	4	CYP, MIT	Scaffold_2: 3255151..3258303
AT5G10230 (ANN7)	*S*. *parvula*	SpUN1172_0010	*SpAnn7*	87%	94%	690	229	8.86	26.63	3	CYP, MIT	Un1172: 0..1530
	*E*.*salsugineum*	Thhalv10014138m.g	*EsAnn7*	89%	96%	954	317	5.45	36.38	4	CYP	Scaffold_2: 3260130..3262434
AT5G12380 (ANN8)	*S*. *parvula*	Sp6g31260	*SpAnn8*	89%	94%	948	315	6.42	35.74	4	CYP, NUC	Ch6_6: 7666290..7668224
	*E*.*salsugineum*	Thhalv10014146m.g	*EsAnn8*	88%	92%	948	315	6.77	35.67	4	NUC, CYP	Scaffold_2: 4077789..4079744

* ID: identity and SI: similarity between Arabidopsis and halophytic species, CDS: Coding Domain Sequence (nucleotide base pair); Size (AA), protein length (Amino Acid residues); pI, theoretical isoelectric point; Mw, Molecular Weight of the amino acid sequence. Subcellular localization: Cytoplasm: CYP; Nuclear: NUC; Mitochondrial: MIT.

**Fig 1:**
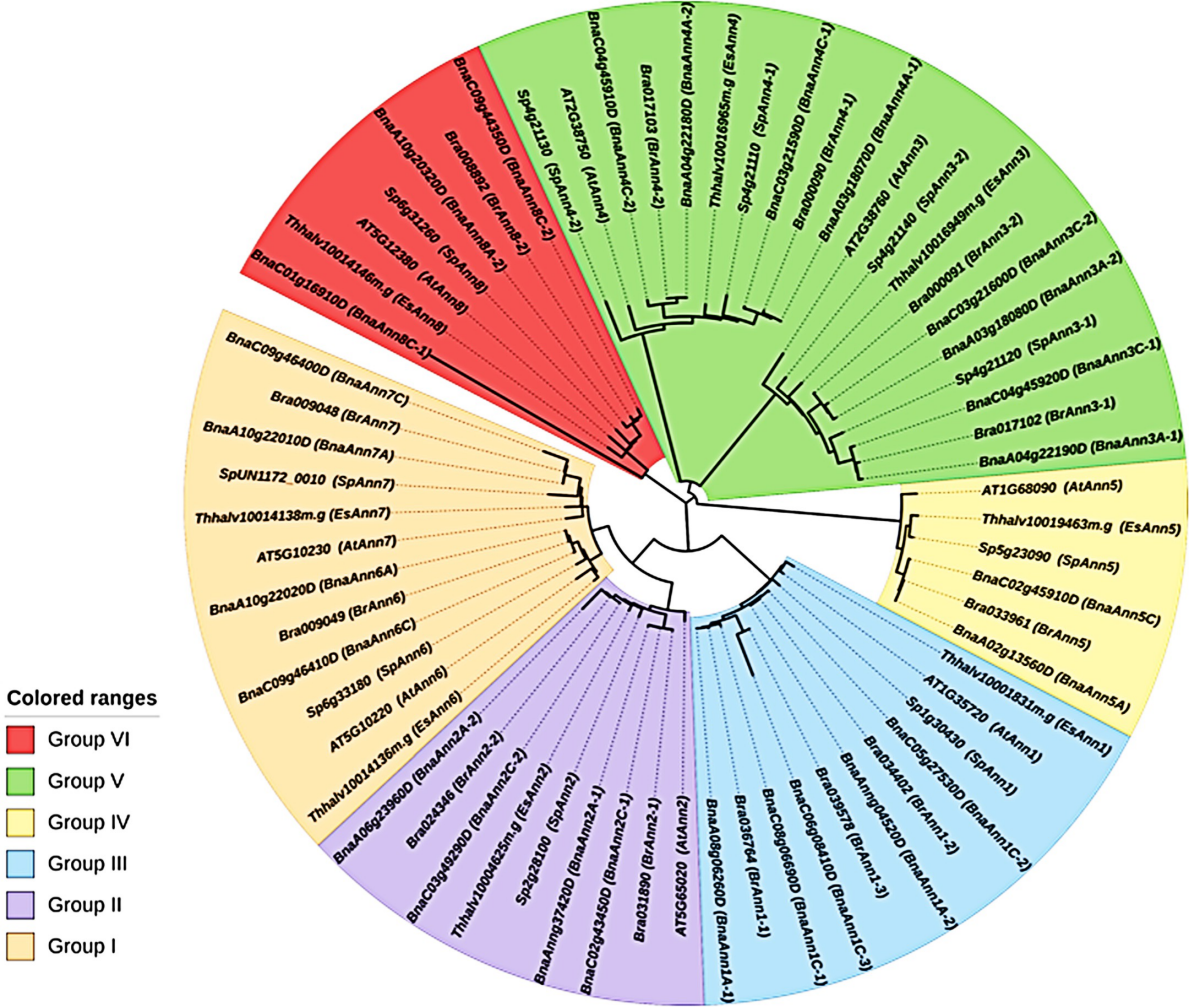
Phylogenetic analysis of SpAnn and EsAnn sequences. The six groupings are represented by the roman letters.

In order to clarify the relationships among the Ann proteins from *A*. *thaliana*, *S*. *parvula*, *E*. *salsugineum* and also *B*. *napus* and *B*. *rapa* as Brassicaceae species, a phylogenetic tree was constructed by aligning protein sequences in the MEGA-X. The results indicated that Ann protein family could be classified into six groups (group I to VI). All *AtAnn* genes were identified to have orthologous genes in *S*. *parvula* and *E*. *salsugineum*. According to these findings, *Ann1*, *Ann2*, *Ann5* and *Ann8* are clustered in groups III, II, IV and VI, respectively. Meanwhile, *Ann6* and *Ann7* are clustered in group I, while *Ann3* and *Ann4* belong to group V (Fig 1).

### Gene structure and conserved motif analysis of *SpAnn* and *EsAnn*

**Fig 2:**
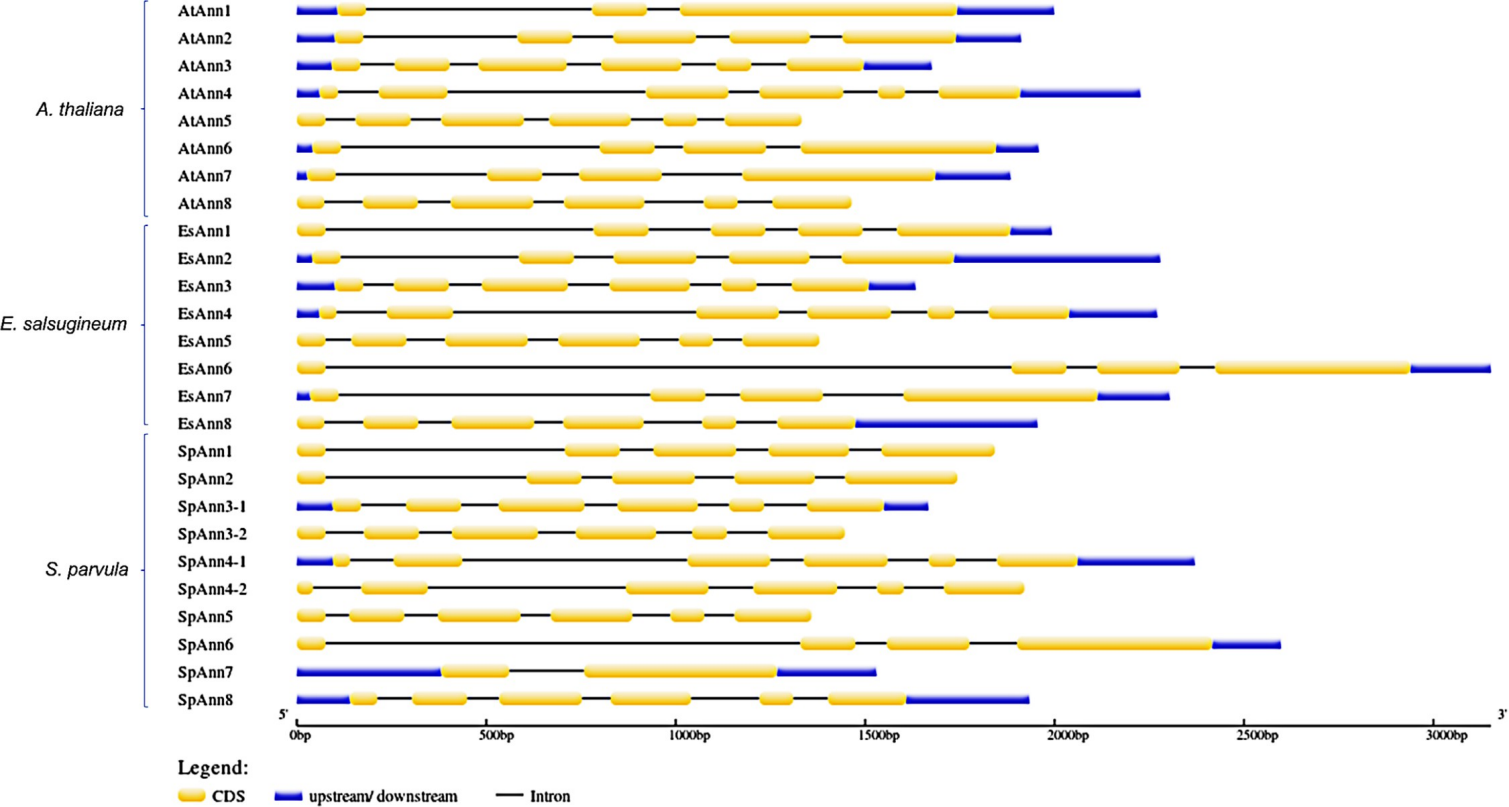
Gene structure of *Ann* genes in *A*. *thaliana*, *S*. *parvula* and *E*. *salsugineum*.

The *SpAnn* and *EsAnn* gene family structures were analyzed and drawn using GSDS v2.0. In the two studied halophytes and *A*. *thaliana*, Es*Ann6* and At*Ann5* have the longest and shortest length of genomic sequence, respectively. The results showed that the majority of homologous *Ann* gene pairs in three studied plants, had the same gene structure. According to these findings, the number of exons per gene ranged from 3 to 6 (regardless of *SpAnn7*). Gene structures are comparable among members of the same phylogenetic group. Six exons and five introns are found in three groups: group IV (*Ann5*), group V (*Ann3* and *Ann4*) and group VI (*Ann8*). Group II (*Ann2*) have five exons and four introns and also group I (*Ann6 and Ann7*) have four exons and three introns. In group III, a little difference was observed in which *Ann1* in *A*. *thaliana* has 3 exons and two introns, but *EsAnn1* and *SpAnn1* have 5 exons and 4 introns in their structure. This data suggests that all of these *Ann* genes have a common ancestor gene and because of their role in development and many biological processes, they have been shown to be conserved during plant evolution (Fig 2).

**Fig 3:**
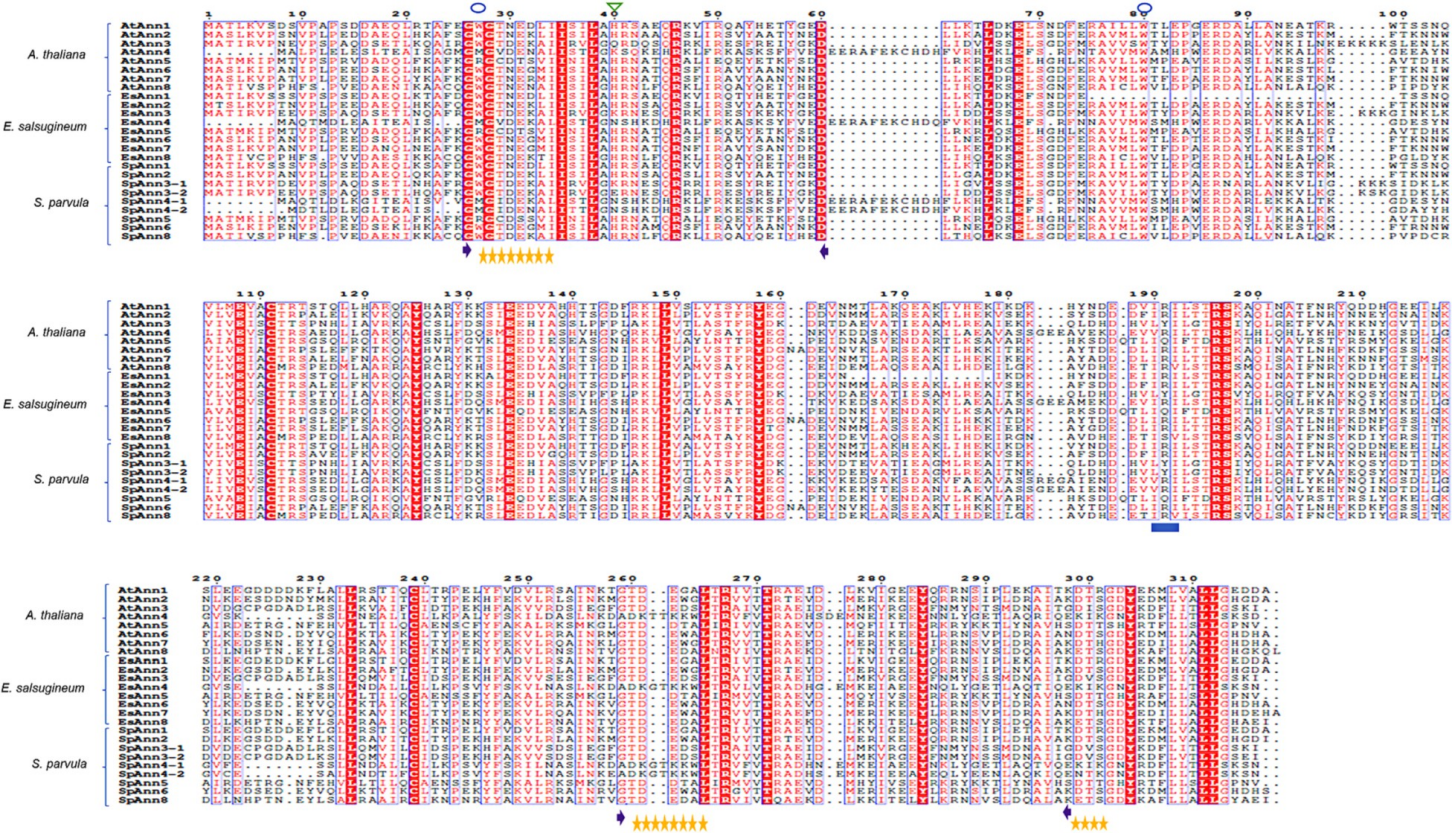
MSA of Ann deduced amino acid obtained by ClustalW in *A*. *thaliana*, *S*. *parvula* and *E*. *salsugineum*. Sequence shading: similarity across groups: blue box; similarity in a group: red box and red character; strict identity: red box and white character. Features: GXGT-38-D/E Ca^2+^-binding sites: purple arrows; conserved tryptophan required for membrane binding: blue circle; His40 key peroxidase residue: green triangle; DXXG putative GTP-binding motif: yellow stars; IRI actin binding motif: blue squares.

All of the Ann proteins, regardless of SpAnn7, were subjected to multiple sequence alignment (MSA) and then the results were used to identify several conserved motifs and residues. Repeats I and IV include Ca^2+^ binding sites (GXGT-38-D/E). All Anns have a conserved tryptophan residues except Ann4 and Ann5, which is required for membrane binding. Total Anns except Ann3 and Ann4 in all studied plants, contain the key peroxidase residue (His40). In repeat IV, each Ann contains ’DXXG,’ a putative GTP-binding motif, although Ann8 and Ann4 in all investigated plants have ’EXXG’ and ’KXXG,’ respectively, instead of ’DXXG’ (SpAnn4-2 has NXXG). In AtAnn5, nonpolar Glycine residue replaced by polar Serine residue in ‘DXXG’. Twelve out of 25 studied Anns in three studied plants, have ‘IRI’, an F-actin binding motif, in their repeat III but it changed to ‘IRV’ in AtAnn8 and SpAnn8 while it changed to ‘ISV’ in EsAnn8. The ‘IRI’ changed to ‘IQI’ and ‘LYI’ in Ann5 and Ann3, respectively (Fig 3).

**Fig 4:**
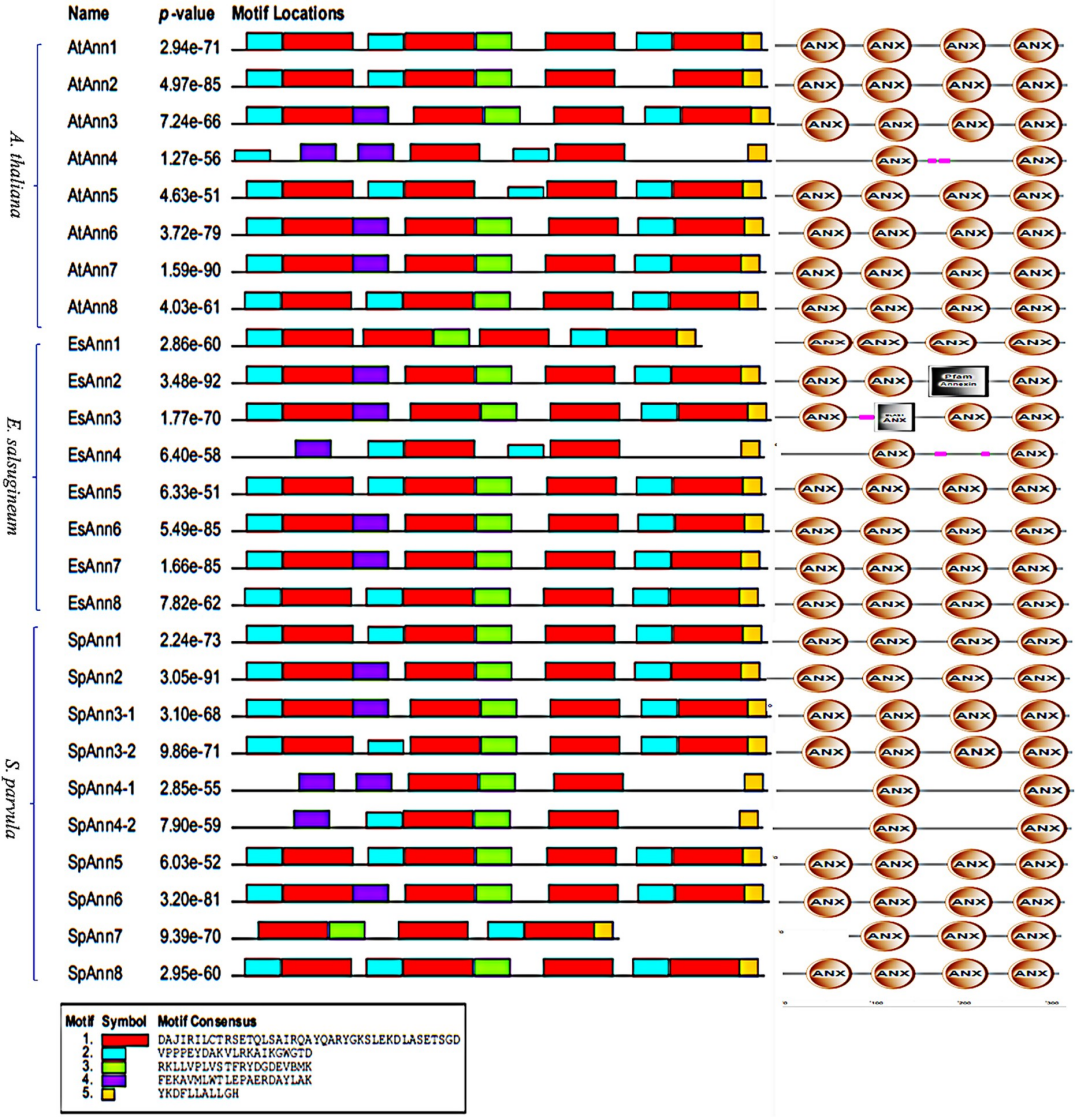
Motif distributions of Ann proteins in *A*. *thaliana*, *S*. *parvula* and *E*. *salsugineum*. Five motifs were identified by MEME tools. On the left, a list of Ann proteins is listed. The various colored boxes symbolize various themes and their locations within each annexin sequence. The key motif sequences are presented at the bottom.

We investigated conserved motifs in 25 Anns of three studied plants to make it easier to compare Ann families. A total of five motifs with 11 to 41 residues were discovered and then were annotated by InterProScan. Motif1 was found in all four annexin repeats as a core sequence and motif5 found in the fourth annexin repeats near the C-terminus (S1 Table). Group I (Ann6 and 7) and group VI (Ann8) members have a similar motif structure in three plants investigated. In group II, AtAnn2 was different from SpAnn2 and EsAnn2 in which the first annexin repeat is missing motif4 in the C-terminus, whereas the second annexin repeat in *AtAnn2* has additional motif2 and its fourth annexin repeat is missing motif2 in the N-terminus. In group III, At and SpAnn1 have a similar motif structure but EsAnn1 lacks motif2 in its N-terminus of second annexin repeat. In group IV, EsAnn5 and SpAnn5 have a similar motif structure but AtAnn5 lacks motif3 in its C-terminus of the second annexin repeat and it has an extra motif2 in its N-terminus of the third annexin repeat. The greatest difference was detected in group V. The members of this group have different motif structures in Ann4 and all of them have two core sequences of annexin repeats. AtAnn3 and EsAnn3 have structures similar to Sp4g21120 (SpAnn3-1) but differ from Sp4g21140 (SpAnn3-2) in that it lacks motif4 in the C-terminus of the first annexin repeat and has an extra motif2 in the second annexin repeat (Fig 4).

### Analysis of cis-acting elements and transcription factor binding sites in *SpAnn* and *EsAnn* gene promoters

**Fig 5:**
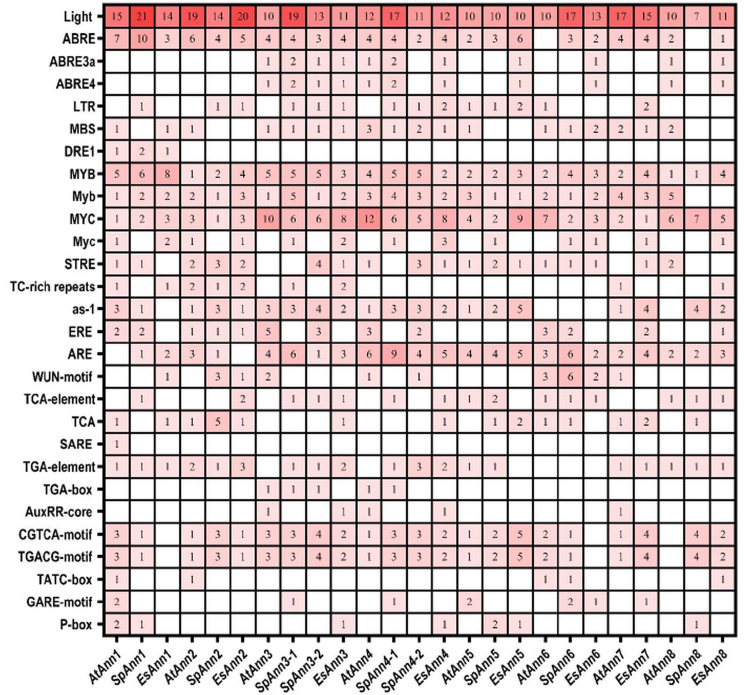
The identified cis-acting elements in *Ann* gene family promoters in *A*. *thaliana*, *E*. *salsugineum* and *S*. *parvula*.

As shown in Fig 5, the promoter of the studied *Ann* genes contains several types of stress responsive elements, including LTR (low temperature responsive elements), DRE1 (dehydration responsive elements), MBS (MYB binding site involved in drought induction), MYB, MYC, STRE (stress responsive elements), TC-rich repeats (defense and stress responsive elements), ERE (elicitor responsive elements), ARE (anaerobic induction responsive element), as-1 (pathogen-induced regulatory elements) and WUN-motif (wound responsive element) (S2 Table). *AtAnns*, *EsAnns* and *SpAnns* contain cis-acting elements involved in phytohormone responses such as ABRE4, ABRE3a and ABRE (ABA responsiveness elements); TCA-element, TCA and SARE (salicylic acid responsive elements); TGA-element, TGA-box and AuxRR-core (auxin responsive elements); CGTCA-motif and TGACG-motif (MeJA responsive elements); TATC-box, GARE-motif and P-box (gibberellin responsive elements). This data indicates that *Ann* genes are highly regulated by multiple cis-acting elements during growth and may play a key role during stress responses.

The results also revealed the presence of several light responsive cis-elements in the *Ann* promoter region of three studied plants. Also, ABA and MeJa related cis-acting elements had the largest number of hormone-responsive cis-elements, respectively. The MeJA- cis-acting elements found in all members of *Ann* gene family except for EsAnn1, EsAnn6 and AtAnn8. All the *Ann* gene promoters contain ABRE except *AtAnn6* and *SpAnn8* (Fig 5). In our study, *SpAnn1* had the largest number of ABRE and also ABRE3a and ABRE4 were more in two halophyte *Ann* genes compared to *A*. *thaliana*. The results also showed that two halophytes have more MYB and Myc cis-acting elements than *A*. *thaliana*. All these data clearly suggest that some *Ann* genes expression may be regulated by salt stress conditions.

**Fig 6:**
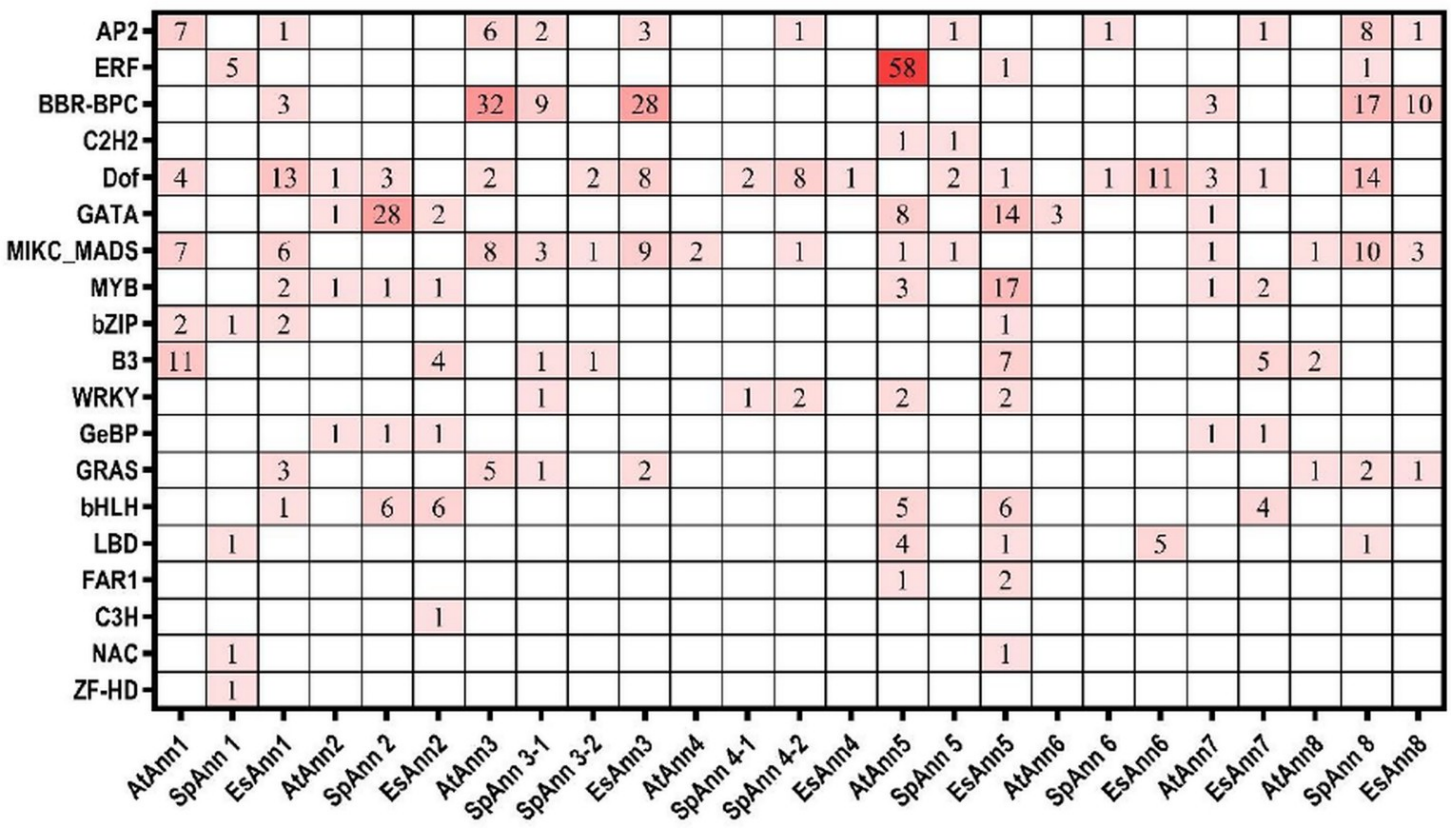
The analysis of TF binding sites in *Ann* gene family promoters in *A*. *thaliana*, *E*. *salsugineum* and *S*. *parvula*.

To obtain information about transcription factors (TFs) of *Ann* genes in three studied plants the putative promoter region of each *Ann* genes was analyzed and various TF families, including AP2/ERF, DOF, GATA, MYB, bZIP and etc. were identified (S3 Table). Additionally, TFBS in the promoter region of studied *Ann* genes were different in terms of number and distribution. For instance, 11 different TFBSs were found in *EsAnn5*, while *EsAnn4*, *AtAnn4*, and *AtAnn6* have only one type of TF binding site. Our results showed that At*Ann5*, Es*Ann5* and Sp*Ann8* have the largest number of TFs and also *AtAnn4*, *EsAnn4* and *SpAnn6* have the least number of binding sites in each studied plant. The largest number of binding sites belongs to BBR-BPC which is followed by DOF, while CCCH zinc finger (C3H) in *EsAnn2* and Zinc Finger HomeoDomain (ZF-HD) in *SpAnn1* with only one binding site have the least number of TFs. Interestingly, the number of GATA TFs found in the *SpAnn2* promoter of *S*. *parvula* (28 GATA TFs) was larger than the total number of GATA TFs found in *E*. *salsugineum* (16 GATA TFs) and also the total number found in *A*. *thaliana* (13 GATA TFs). It is noteworthy that, there are six bHLH TF binding sites in *Ann2* of both halophytes, but none in *Ann2* of *A*. *thaliana* (Fig 6). This indicates that members of the GATA, DOF and bHLH transcription factor families could possibly regulate the *Ann* gene in extremophile plants, which needs further experimental verification.

### Nonsynonymous/Synonymous substitution rate ratio (Ka/Ks)

The evolutionary constraints affecting the *Ann* gene family were determined using the Ka, Ks, and the Ka/Ks value. Differences between the aligned sequences may result in differences in amino acids (nonsynonymous changes) or leave the amino acids unchanged (synonymous changes) and counting them up will provide us an idea of how much the sequence has been changed. The ratio of the number of nonsynonymous substitutions per nonsynonymous site (Ka) to the number of synonymous substitutions per synonymous site (Ks) determine the selective forces acting on the protein. The Ka/Ks value can be greater than one, less than one or equal to one, indicating positive, purifying or neutral selection, respectively. Positive selection fixes beneficial variations, while purifying selection removes deleterious variations, whereas neutral selection is neither beneficial nor detrimental on a set of homologous protein-coding genes [40, 41]. The majority of *Ann* genes had a Ka/Ks value less than one, which implies that these genes during the evolution have been subjected to purifying selection. Nevertheless, the Ka/Ks value of *AtAnn2/SpAnn2* which was greater than one signifies positive selection (S4 Table).

### *Ann* gene expression patterns

**Fig 7:**
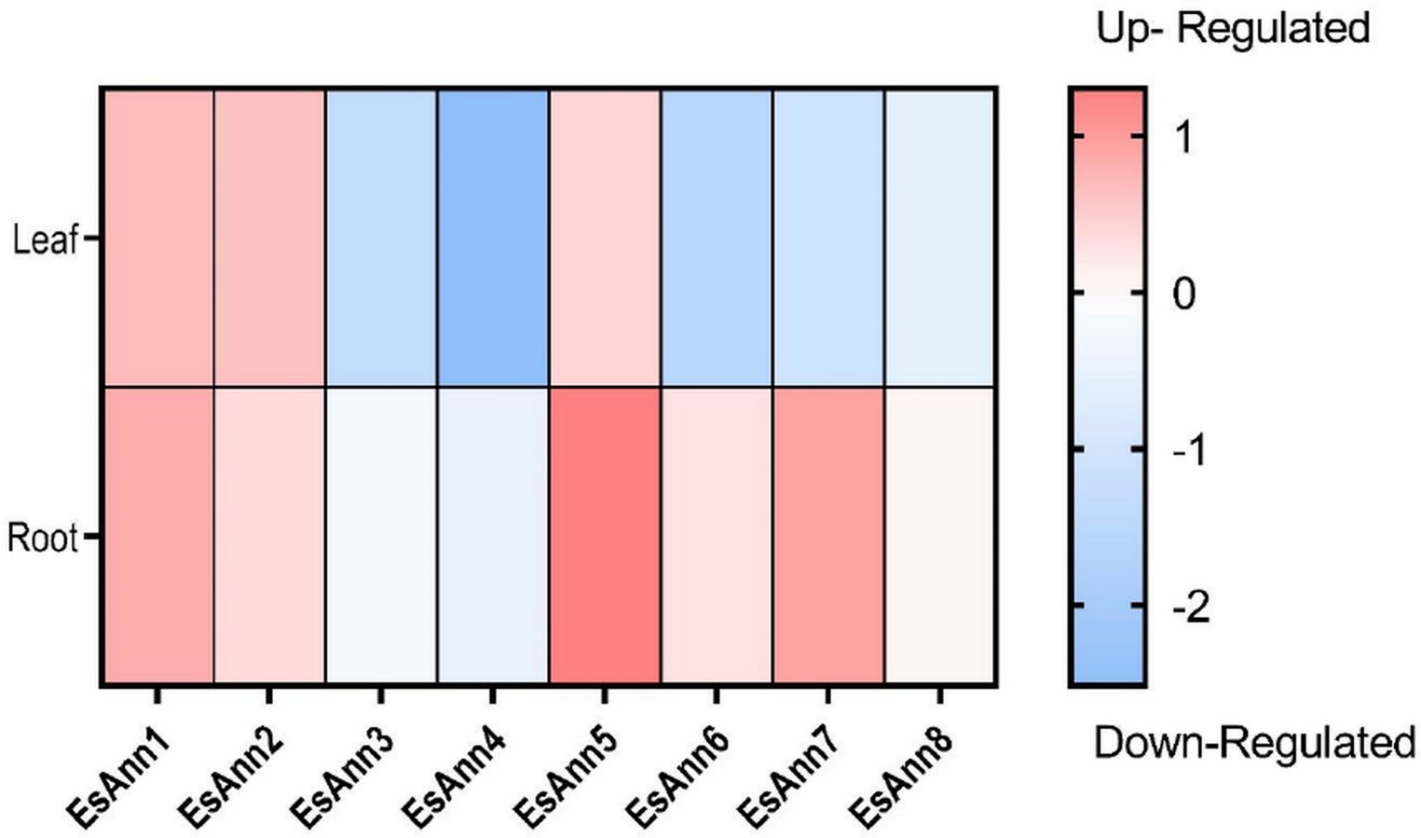
Expression pattern of *E*. *salsugineum Anns* under salt stress treatments. The gradient bar indicates the expression value of log2FC. Thirty days after germination, *E*. *salsugineum* seedlings were treated with 300 mM NaCl and after 24 h, leaves and roots were separately collected.

To reveal the responses of the *E*. *salsugineum Ann*s to salt stress, we investigated their levels of expression [38] (S6 Table). The results indicated that in leaves, *EsAnn*1, 2 and 5 were up-regulated (with 0.69, 0.61 and 0.42 log2 fold change respectively), while *EsAnn*3, 4, 6 and 8 were down regulated (with -1.38, -2.51, -1.5 and -1.12 log2 fold change respectively). In roots, *EsAnn*1, 2, 5, 6 and 7 were up-regulated (with 0.83, 0.36, 1.2, 0.30 and 0.94 log2 fold change) and others had weak differential expression (Fig 7).

**Fig 8:**
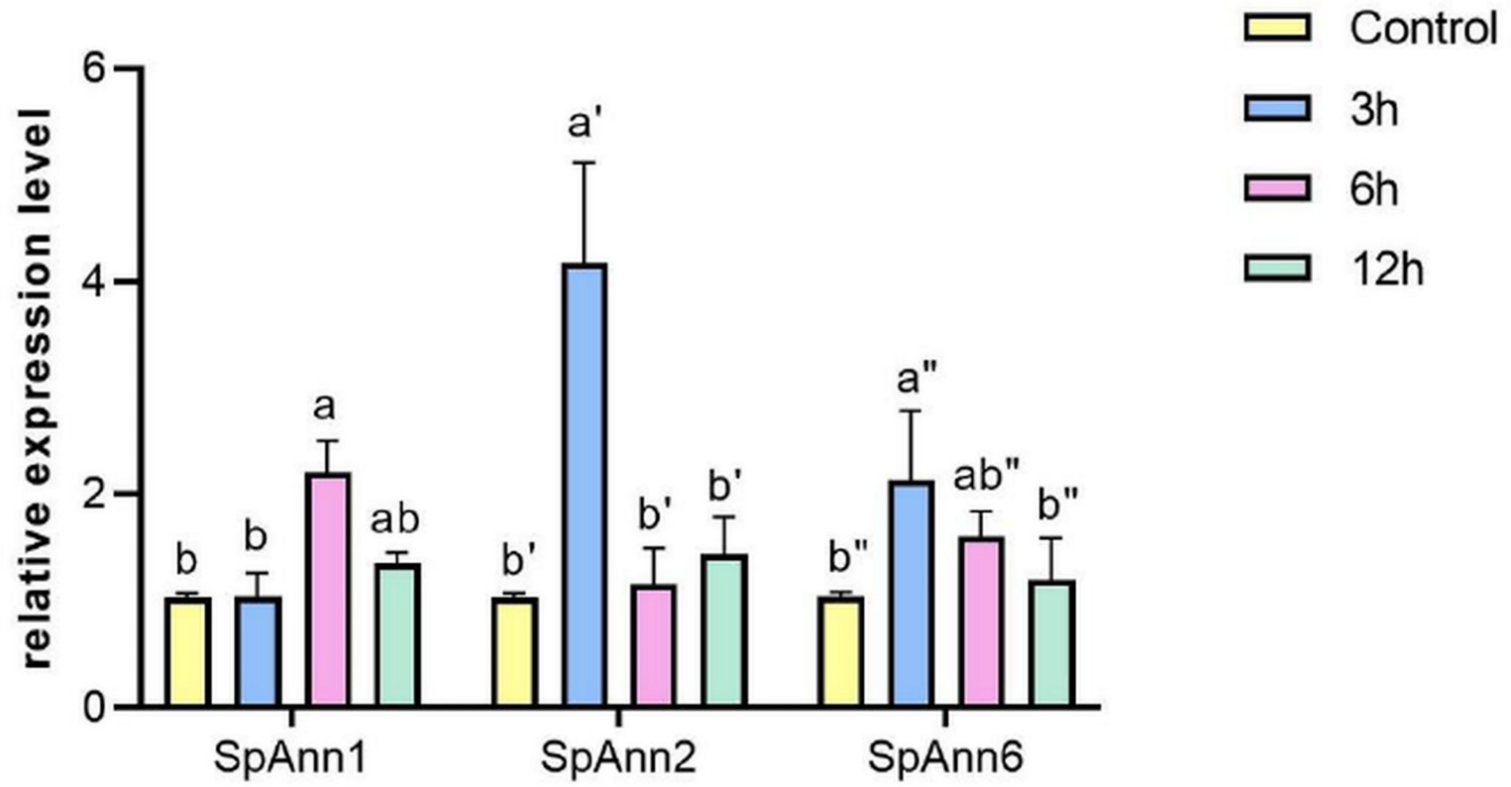
Expression of selected of *S*. *parvula Anns* under Salt stress treatments. Three-weeks old seedlings were treated with 200 mM NaCl for 3 h, 6 h, and 12 h. The expression level was normalized against Act 7 gene. Different letters represent statistically significant differences (P≤0.05).

Based on cis-acting elements and TFs analysis as well as previous studies, three *Ann* genes (*SpAnn*1, 2, and 6) in *S*. *parvula* were selected to confirm the *in-silico* results using qRT-PCR. We found that compared to the control, the studied genes were up-regulated at least at one of the salt treatment time points (P≤0.05). In comparison with control conditions, in 6 h after salt stress, *SpAnn1* gene expression was significantly up-regulated (with 2.20 relative expression level). *SpAnn2* gene expression was highly induced 3h after salt stress (with 4.17 relative expression level) and significantly repressed 6 and 12 h later (with 1.16 and 1.44 relative expression level respectively). *SpAnn6* gene expression was significantly increased 3 h after salt stress (with 2.13 relative expression level) compared to 6 h and 12 h (Fig 8). Analysis of candidate gene expression (*SpAnn*1, 2, and 6) in root of *S*. *parvula* was retrieved from Li et al. [39] (S7 Table) research. Based on the results, *SpAnn6* showed the greatest difference in 3 h after salt treatment. However, weak changes (range from -0.447 to +0.496) was detected in root (S2 Fig). These findings imply the role of mentioned genes in the salt stress response in the shoot of *S*. *parvula* (Fig 8).

## Discussion

Plants are exposed to various abiotic stresses, which influence crops growth and development. *Ann* gene family are considered to play important role in abiotic stress tolerance [5, 14]. Several *Ann* genes have been discovered in plants, including eight in Arabidopsis [6], ten in rice [10], 25 in wheat [11] and 26 in B. napus [9] but up to now there hasn’t been reported any study about the *Ann* gene family in extremophile plants. In this study, eight and ten *Ann* genes found in the *E*. *salsugineum* and *S*. *parvula* genomes, respectively. Based on the studies of Oh et al. [42] who worked on genome structural variations in *A*. *thaliana* and *S*. *parvula*, it was found that shared tandem duplication happened in *SpAnn3-1* and *SpAnn3-2* and also *SpAnn4-1* and *SpAnn4-2*. The theory that gene duplications often occur as tandem duplications is supported by the similar amino acid sequences, same chromosome localization and short distance between these predicted genes [43] and gene duplication events may have increased the number of *Ann* genes in *S*. *parvula* which is in agreement with Clarks et al. [44] findings. *Ann* genes were found on five chromosomes and five scaffolds in *S*. *parvula* and *E*. *salsugineum*, respectively. We were unable to map genes into the chromosomes of two extremophile plants due to limitations in sequencing quality and assembly methods. These proteins were classified into six groups (Fig 1), which was consistent with a previous report on the Brassicaceae species [9]. One of the key factors related to Ann protein’s functions is its subcellular distribution profile [14]. Plant Ann proteins are located in different sites, including cytoplasm and plasma membrane [12, 45], nucleus [20, 46], chloroplast [47] and tonoplast [48]. EsAnns and SpAnns were predicted to have three subcellular localization sites: the cytoplasm, nucleus and mitochondria. Our findings are in partial agreement to those obtained before.

In our study, the majority of homologous *Ann* gene pairs in three studied plants, had similar gene structures. All 8 members of *Ann* genes in *A*. *thaliana* were identified to have orthologous genes in *S*. *parvula* and *E*. *salsugineum*. The presence of some conserved motifs and the four repeats in the At, Sp and Es Ann amino acid sequences (Figs 3 and 4), suggests that all of these *Ann* genes have a common ancestor gene. plant Ann have some conserved motifs and residues which are important for its activity including Ca^2+^ binding site, conserved tryptophan for membrane binding, His40 as key peroxidase residue, ATP/GTP binding motif and F-acting binding motif. The findings agree with previous researches [9, 10, 49] which show that *SpAnns* and *EsAnns* are members of a multi-gene family of *Anns* that are conserved due to their important biological functions. However, there are some differences between the two halophytes and *A*. *thaliana* as a glycophyte, which might be linked to their tolerance to abiotic stresses. For instance, comparing AtAnn2 with SpAnn2 and EsAnn2 shows that due to the replacement of Alanine 85 by proline 85 in AtAnn2 (green arrow 1), the first annexin repeat misses motif4 in the C-terminus and the second annexin repeat has additional motif2. Moreover, the fourth annexin repeat in AtAnn2 misses motif2 in the N-terminus through the replacement of Alanine 254 by Serine (green arrow 2) (S1 Fig).

He et al. [9], investigated the Ka/Ks value between paralogous *Ann* gene pairs in *A*. *thaliana*, *B*. *rapa*, *B*. *oleracea* and *B*. *napus*. They found that most of the genes experienced purifying selection but gene pair of *BrAnn2-2/BnaAnn2A-2* (*Bra024346/BnaA06g23960D)* experienced positive selection. In our investigation, *AtAnn2*/*SpAnn2* experienced positive selection whereas the others experienced purifying selection.

Based on cis-acting elements analysis, responses to many biotic and abiotic stresses are associated with motifs found in the promoter regions of the *Anns*, including ABRE, MBS, DRE1, LTR, MYB, MYC, STRE, TC-rich repeats, as-1, ERE and WUN-motif, etc. (Fig 5). The most abundant cis-elements were the light responsive, which were followed by MYC and ABRE. Furthermore, ABREs were the most numerous among hormone responsive cis-elements. Among the stress related elements, ABRE, ABRE3a, ABRE4, MBS, DRE1, Myb, MYB, Myc, MYC are supposed to be associated with plants’ responses to salt stress [50]. It is to be noted that the promoter regions of the two halophytes exhibit a greater number of MYB, Myc and ABRE cis-elements than *A*. *thaliana* as a glycophyte. The results suggest that *Anns* expression may be controlled by hormones, abiotic and biotic stress conditions which implies its role in growth, development and stress tolerance.

In this study, only *Ann1*, in all three investigated plants, had DRE1 in its promoter. Previous studies supported its role in abiotic stresses [51, 52]. Furthermore, all mentioned *Ann1* had numerous ABRE that have been shown to improve abiotic tolerances [53–55]. Among all the studied *Anns*, only *SpAnn1* had a Zinc Finger Homeo Domain (ZF-HD) and it also had a NAC TF binding site in its promoter, which its role in drought and salt tolerance has been confirmed [56–58]. According to cis-acting elements analysis in our study, the expression patterns of selected *SpAnn1* as well as *EsAnn1* were investigated, which showed their upregulation in response to NaCl treatment. The results imply *Ann1*’s role in abiotic stress tolerance, which is compatible with previous studies [19, 45, 59].

Transcription factors (TFs) participate in regulating a wide variety of target genes that are responsible for plant adaptation and tolerance [60]. A variety of TF families, including AP2/ERF, NAC, WRKY, MYB and bZIP, have been reported to be linked to stress responses [50]. More than 1500 TFs were identified in the Arabidopsis genome sequence [61] and among these TFs, HSFA2, bZIP24, WRKY33, MYB41, ANAC042 and C2H2-type zinc fingers have been suggested to play critical roles in response to salt and osmotic stress [62]. We investigated the regulation of TFs under the expression of *Ann* genes in *A*. *thaliana*, *E*. *salsugineum* and *S*. *parvula* and found some differences in the TF binding site types, number and distribution (Fig 6). The two halophytes have a greater number of DNA binding with one finger (DOF) compared to *A*. *thaliana*. Earlier studies verified DOF involvement in salt tolerance [63]. According to the genes formerly mentioned in *E*. *salsugineum* [38], it could be deduced that *Anns* are related to TF genes that are involved in response to salt stress, including *SpAnn2* (DOF1), *SpAnn4-2* (DOF1) and *SpAnn6* (DOF1), *SpAnn8* (DOF1, DOF2) in *S*. *parvula* and *EsAnn3* (DOF2), *EsAnn5* (DOF1) and *EsAnn6* (DOF1, DOF2) in *E*. *salsugineum*.

Interestingly, in our investigation, GATA TFs were found in only one member of *Ann* genes promoter in the *S*. *parvula* (*SpAnn2*), but it was either greater than the total numbers of GATA in *E*. *salsugineum* or greater than that of *A*. *thaliana*. Gupta et al. [64] investigated GATA gene expression profiles in rice in response to salinity, drought and ABA treatments and found that some of the GATA genes have higher transcript levels in the salt tolerance genotype as compared to the salt sensitive variety. They mentioned that OsGATA1 and OsGATA10 might mediate abiotic stress responses and signaling. Zhao et al. [65], indicated that the SlGATA17-overexpressing tomatoes were more drought tolerant. Other studies also confirmed that GATA transcription factors promote abiotic tolerance [66, 67]. It is noteworthy that *Ann2* in both halophytes had six bHLH TF binding sites while *A*. *thaliana* did not. The bHLH TFs enhance plant’s tolerance to abiotic stresses [68–71] for instance, in *A*. *thaliana*, AtbHLH028, AtbHLH92 and AtbHLH122 and also OsbHLH035, OsbHLH062 and OsbHLH068 in *Oryza sativa* are reported to participate in salt stress responses [72]. Based on TFs analysis, the expression patterns of selected *SpAnn2* and also *EsAnn2* were investigated, which revealed that they were upregulated in response to NaCl treatment. Several studies have also reported *Ann*2’s role in abiotic stress responses [20, 73].

According to RNA-seq data from Oh et al. [42], *Ann6* in *S*. *parvula* compared to the *A*. *thaliana* homolog in basal (control) condition, shows 4.12- and 5.53-fold higher expression in roots and shoots, respectively (S8 Table). Besides, Harbaoui et al. [74] reported that overexpressed Arabidopsis plants with *Triticum durum TdAnn6* improved salt and osmotic stress tolerance. In our investigation, NaCl treatment increased the expression level of the selected *SpAnn6* gene in *S*. *parvula* shoots.

The results showed significant upregulation of EsAnn1, 5 and 7 in the roots of *E*. *salsugineum* seedlings which were treated with 300 mM NaCl and collected after 24 h [38] while in *S*. *parvula’*s seedlings treated with 175 mM NaCl and collected after 3 h, 24 h, 48 h [39], candidate genes (Ann1,2 and 6) showed weak expression changes (range from -0.447 to +0.496). These differences in expressions could be attributed to the difference in NaCl concentration (175mM vs 300mM) and different seedling growth phase (4 days vs 30days after germination) in two halophytes. He et al. [9] found that most of Ann genes in group of *Ann1*, *2*, *3* And *4* were up-regulated under salinity and PEG stress in roots in *Brassica napus*. Some studies have shown that *AtAnn1* and *AtAnn2* regulated the growth and development of roots. Laohavisit et al. [15] demonstrated that root cell adaptation to salinity is impaired in the loss-of-function mutant of *AtANN1*. Wang et al. [75] also showed that AtANN1 and AtANN2 are important in post-phloem sugar transport to the root tip, which influences photosynthetic rates in cotyledons. Ectopic expressed Ann6 of cotton in Arabidopsis made the root of transgenic plants longer due to the enlargement of root cells, without increasing the root cell number, through its interaction with actin 1 [76]. We also found that *EsAnn1*, *2* and *5* upregulated in *E*.*salsugineum*’s leaves under salt stress treatment. These upregulations, as well as the expression pattern of three selected *SpAnn* genes in the shoot (*SpAnn*1, 2 and 6) support *Anns’* role in salt stress tolerance. The aforementioned expression profiles agree with cis-acting elements and TFs analysis.

Although upregulation of all Arabidopsis *AtAnns* except for *Ann2* and *Ann3* was observed in Arabidopsis salt induced *AtAnns* [77], we found that *Ann2* in *S*. *parvula* was significantly upregulated in salt stress conditions. The divergent expression patterns of the two studied halophytes compared to *A*. *thaliana* as a glycophyte reveals that *Ann* genes in halophytes might change regulatory mechanisms that contribute to salt tolerance.

## Conclusion

In this study, the *S*. *parvula* and *E*. *salsugineum* genomes were investigated in terms of *Ann* genes and ten and eight *Ann* genes were identified, respectively. Although the majority of homologous *Ann* gene pairs in *S*. *parvula*, *E*. *salsugineum* and *A*. *thaliana* had the same structure, conserved motifs and residues, the regulatory mechanisms (cis-acting elements and transcription factors) in halophytes are more salt responsive in compare to *A*. *thaliana* as a glycophyte. We chose three *SpAnn* genes (*SpAnn*1, 2 and 6) in *S*. *parvula* based on promoter region analysis as well as previous studies, which upregulated in response to NaCl treatment in the shoot. The expression patterns of *E*. *salsugineum* under salt stress supported *Anns’* role in salt stress tolerance. The divergent expression patterns of the investigated plants showed that *SpAnn2* was upregulated under salt stress conditions, while *AtAnn2* was not. Further investigation of the roles of candidate *Ann* genes in abiotic stresses, would be of great interest.

## Supporting information

S1 Table Gene ontology (GO) terms annotation of five motifs in AtAnn, SpAnn and EsAnn proteins Identified by MEME tools. (XLSX)

S2 Table List of cis-acting elements present in *AtAnn*, *SpAnn* and *EsAnn* gene promotors. Analyzed by PlantCARE.
(XLSX)

S3 Table List of TF binding sites in AtAnn, SpAnn and EsAnn gene promotors. (XLSX)

S4 Table Ka/Ks ratios of the *Ann* genes. (XLSX)

S5 Table List of primers used in this study. (XLSX)

S6 Table RNA-seq data of expression levels of *E*. *salsugineum Anns* under salt stress treatments. (XLSX)

S7 Table RNA-seq data of expression levels of *S*.*parvula* annexins under salt stress treatments. (XLSX)

S8 Table RNA-seq results comparing expression strengths of homologs between *S*. *parvula* (Sp) and *A*. *thaliana* (At). (XLSX)

S1 Fig MSA of AtAnn2, EsAnn2 and SpAnn2 deduced amino acid obtained by ClustalW. (TIF)

S2 Fig Heatmap presenting expression profile of *SpAnn1*, *2* and *6* in root of *S*. *parvula* under salt stress at 3h, 24h and 48h. (TIF)
