# Supplementary figures and images for: Genome-wide analysis of annexin gene family in Schrenkiella parvula and Eutrema salsugineum suggests their roles in salt stress response
Source: PLoS One. 2023 Jan 18;18(1):e0280246. doi: 10.1371/journal.pone.0280246 (PMC9847905; doi:10.1371/journal.pone.0280246)

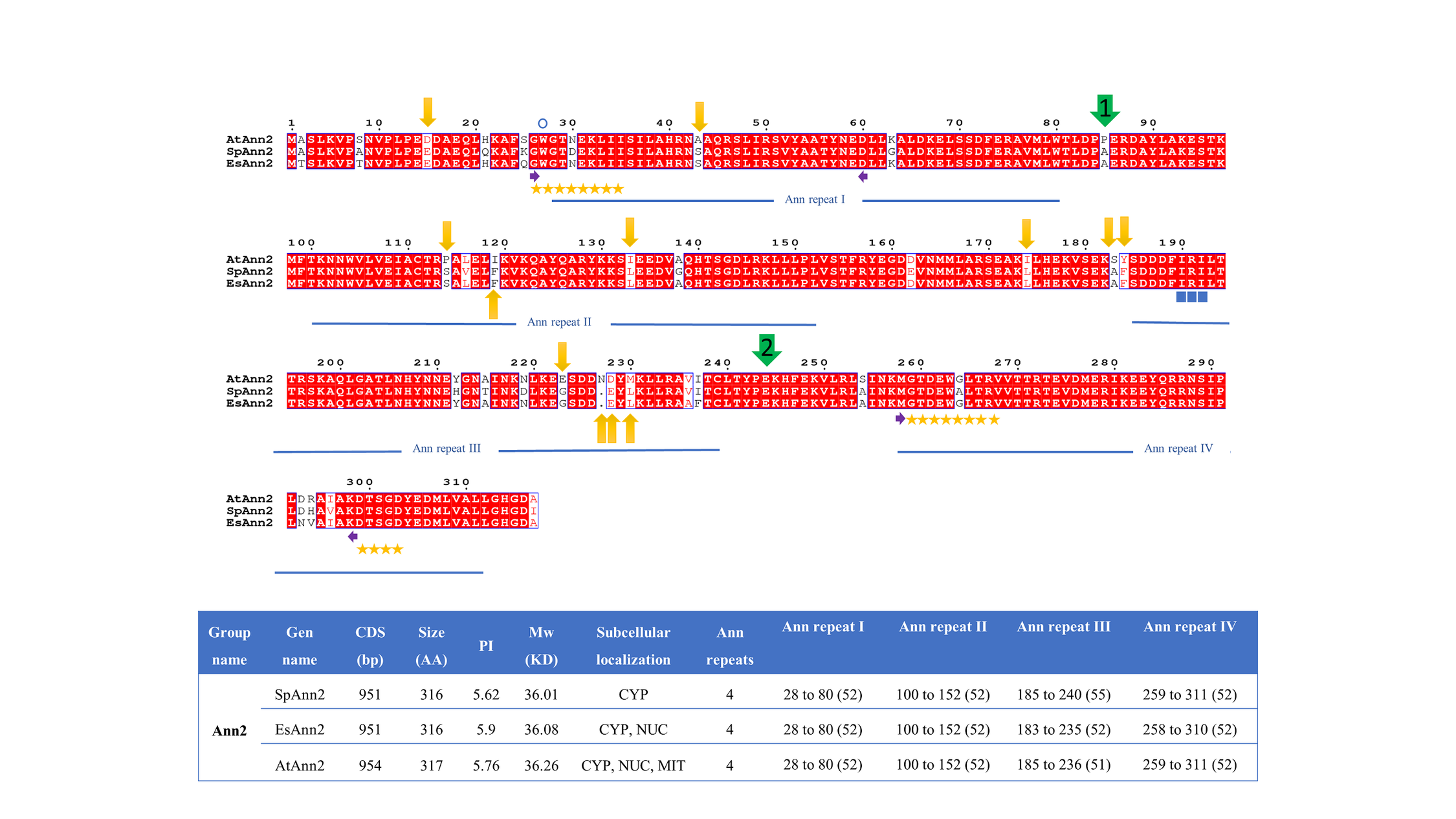

Supplement: S1 Fig — (TIF) [file pone.0280246.s009.tif]

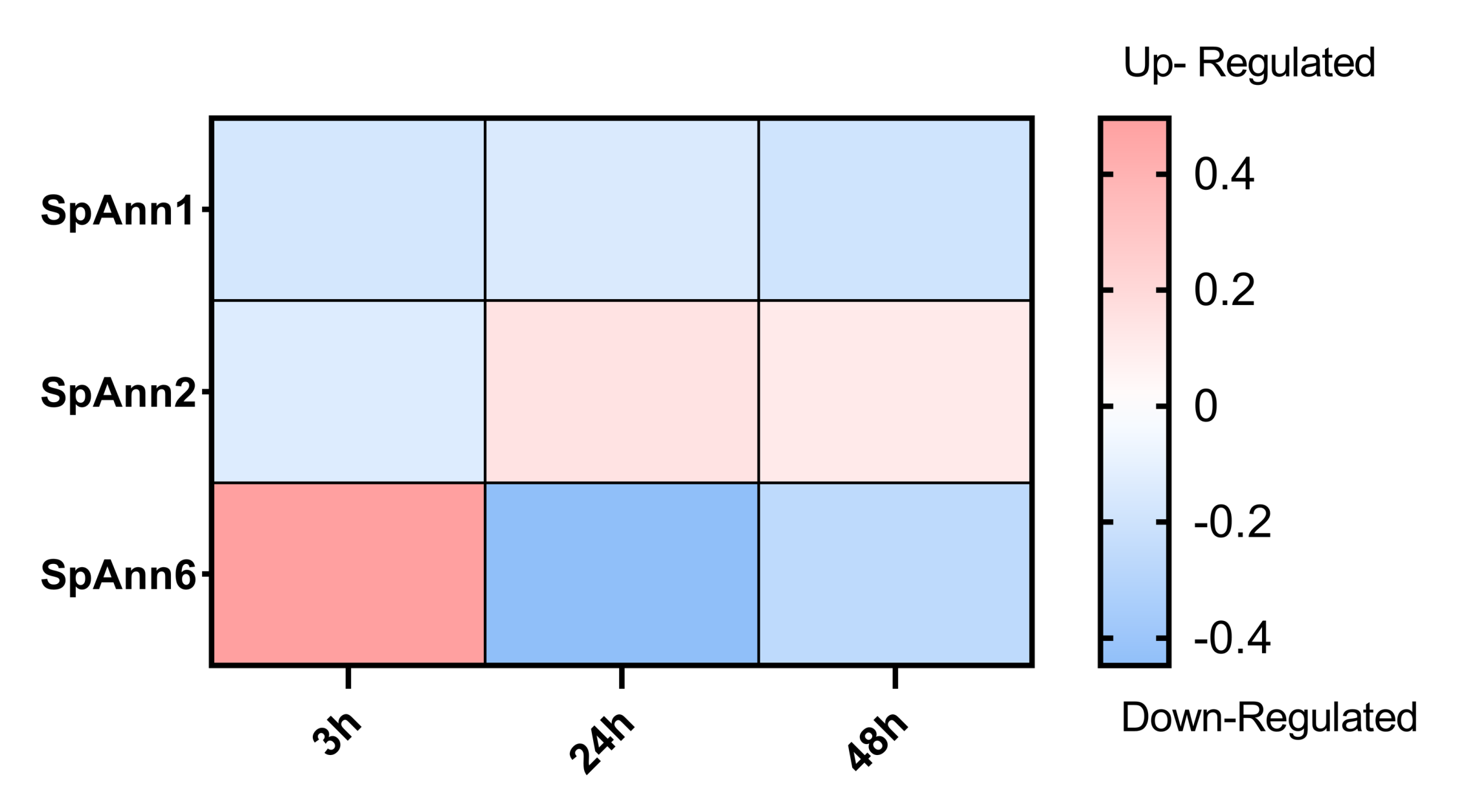

Supplement: S2 Fig — (TIF) [file pone.0280246.s010.tif]
